# Next-generation sequencing reveals altered gene expression and enriched pathways in triple-negative breast cancer cells treated with oleuropein and oleocanthal

**DOI:** 10.1007/s10142-023-01230-w

**Published:** 2023-09-14

**Authors:** Paraskevi Karousi, Christos K. Kontos, Panagiota Papakotsi, Ioannis K. Kostakis, Alexios-Leandros Skaltsounis, Andreas Scorilas

**Affiliations:** 1 Department of Biochemistry and Molecular Biology, Faculty of Biology, National and Kapodistrian University of Athens, Athens, Greece; 2 Pharmagnose S.A., 57km National Road Athinon-Lamia, Inofyta, Greece; 3 Division of Pharmaceutical Chemistry, Department of Pharmacy, National and Kapodistrian University of Athens, Athens, Greece; 4 Division of Pharmacognosy & Natural Products Chemistry, Department of Pharmacy, National and Kapodistrian University of Athens, Athens, Greece

**Keywords:** Transcriptomics, RNA sequencing (RNA-seq), Signaling pathways, *Olea europaea*, Natural products, Novel therapeutic strategies

## Abstract

Triple-negative breast cancer (TNBC) is a subtype of breast cancer characterized by poor prognosis and limited treatment options. Oleuropein and oleocanthal are bioactive chemicals found in extra-virgin olive oil; they have been shown to have anti-cancer potential. In this study, we examined the inhibitory effects of these two natural compounds, on MDA-MB-231 and MDA-MB-468 TNBC cell lines. The human TNBC MDA-MB-231 and MDA-MB-468 cell lines were treated with oleuropein or oleocanthal at ranging concentrations for 48 h. After determining the optimum concentration to reach IC50, using the sulforhodamine B assay, total RNA was extracted after 12, 24, and 48 h from treated and untreated cells. Poly(A)-RNA selection was conducted, followed by library construction and RNA sequencing. Differential gene expression (DEG) analysis was performed to identify DEGs between treated and untreated cells. Pathway analysis was carried out using the KEGG and GO databases. Oleuropein and oleocanthal considerably reduced the proliferation of TNBC cells, with oleocanthal having a slightly stronger effect than oleuropein. Furthermore, multi-time series RNA sequencing showed that the expression profile of TNBC cells was significantly altered after treatment with these compounds, with temporal dynamics and groups of genes consistently affected at all time points. Pathway analysis revealed several significant pathways associated with TNBC, including cell death, apoptotic process, programmed cell death, response to stress, mitotic cell cycle process, cell division, and cancer progression. Our findings suggest that oleuropein and oleocanthal have potential therapeutic benefits for TNBC and can be further investigated as alternative treatment options.

**Supplementary Information:**

The online version contains supplementary material available at 10.1007/s10142-023-01230-w.

## Introduction

Uncontrolled growth and spread of abnormal cells in the breast tissue characterize the complicated and variable disease known as breast cancer (BrCa). It is the most prevalent type of cancer in women worldwide and a serious public health issue (Ferlay et al. 2021). BrCa has a complex etiology, involving both hereditary and environmental factors (Harbeck and Gnant 2017; Harbeck et al. 2019; Loibl et al. 2021). Based on distinct gene expression patterns, several molecular subtypes of BrCa have been discovered. These subtypes vary in clinical behavior and responsiveness to therapy (Cancer Genome Atlas 2012; Sung et al. 2021).

The most common subtypes of BrCa include hormone receptor-positive, HER2-positive, and triple-negative BrCa (TNBC) (Polyak 2011; Testa et al. 2020). TNBC is a particularly aggressive kind of BrCa with a worse prognosis and a higher chance of recurrence than other subtypes. The effective treatment choices for TNBC are very limited, considering that a lot of hormonal treatments and targeted medications that target the HER2 receptor have been unsuccessful so far. Chemotherapy is thus the cornerstone of TNBC treatment (Lehmann et al. 2011).

A key area of research is the generation of BrCa medicines that are both safe and effective (Waks and Winer 2019), with a rising focus on natural products and their derivatives. A key element of the Mediterranean diet, which has been linked to a lower incidence of cancer, is extra-virgin olive oil (EVOO) (Vatansever et al. 2022). Oleuropein and oleocanthal are two bioactive compounds found in EVOO that have been shown to have anti-cancer potential (Jimenez-Lopez et al. 2020; Silenzi et al. 2020). More specifically, oleuropein, a secoiridoid glycoside present in EVOO, has been shown to have anti-cancer properties in a range of cancer cell lines, including BrCa cell lines (Asgharzade et al. 2020). This compound causes cell cycle arrest and apoptosis and is an intriguing option for the treatment of BrCa due to its anti-inflammatory, antioxidant, and anti-angiogenic effects (Fabiani et al. 2012; Rishmawi et al. 2022). The phenolic component oleocanthal has been shown to have powerful anti-inflammatory and anti-cancer properties (Parkinson and Keast 2014; Rigacci and Stefani 2015). Studies have demonstrated that oleocanthal prevents cancer cells from proliferating, migrating, and invading, possibly via downregulating COX-2 and inhibiting NF-κB signaling (Akl et al. 2014; Siddique et al. 2020). Several studies recently examined the effects of oleuropein and oleocanthal on BrCa cells. According to one of them, oleuropein affects the expression of genes involved in cell cycle regulation and apoptosis, which decreases the proliferation and increases apoptosis of TNBC cells (Hassan et al. 2014). In another study, oleocanthal was found to affect the expression of genes linked to BrCa cell survival and proliferation (Qusa et al. 2021).

Next-generation sequencing (NGS) is a powerful method, suitable for examining the molecular impact of drug treatment on cancer cells (Kim et al. 2021; Kushwaha et al. 2022; Li et al. 2021). Transcriptomics analyses allow the detection of differences in gene expression patterns in response to therapy, providing significant new insights into the underlying mechanisms of action. In this study, we used transcriptome analysis to examine the impact of oleuropein and oleocanthal on the expression profiles of TNBC cells. We propose that oleuropein and oleocanthal can alter the expression of genes implicated in the progression of TNBC. Our results provide a thorough understanding of the molecular processes underlying the anti-cancer impact of oleuropein and oleocanthal on TNBC cells and highlight the potential of these two bioactive natural products as novel treatment options.

## Methodology

### Oleocanthal synthesis

**Fig. 1:**
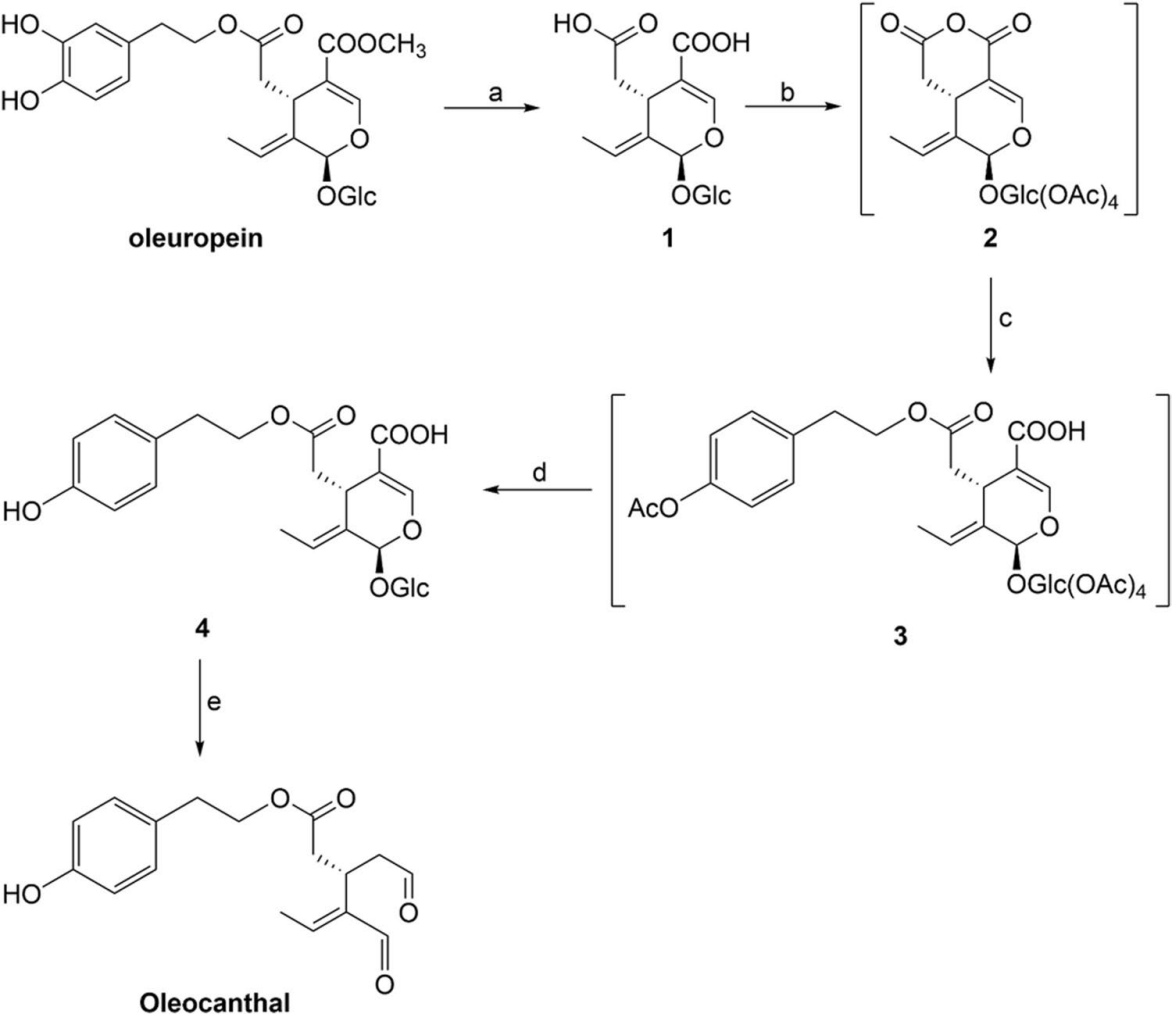
Reaction conditions: (**a**) NaOH, H_2_O, rt, 24 h; (**b**) Ac_2_O, py, rt, 24 h; (**c**) 2-phenylethanethiol, DMAP, Et_3_N, CH_2_Cl_2_, Ar, 0 °C to rt, 20 h; (**d**) Et_2_NH, MeOH, rt; (**e**) HCl 1N, 28 °C

Our study primarily focused on developing a scalable procedure for the synthesis of oleocanthal. To address challenges such as lengthy steps, purification issues, and overall yield, we utilized a previously described protocol from our research group for synthesizing oleocanthal analogs (Sarikaki et al. 2020). In summary, oleuropein was hydrolyzed in a single step to obtain the corresponding oleoside (**1**), which was purified by fast centrifugal partition chromatography (FCPC) (Fig. 1). Treatment of oleoside with acetic acid anhydride resulted in mixed anhydride **2**, which, upon reaction with acetylated tyrosol, yielded carboxylic acid **3**. Deprotection of **3**, upon treatment with diethylamine, provided the glycosylated carboxylic acid **4**, which was then subjected to acidic hydrolysis resulting in oleocanthal. Previously, the final step was accomplished through enzymatic hydrolysis with β-glucosidase. However, the new protocol is straightforward and more convenient, with a higher overall yield (30.4%) (Supplementary Materials and Methods). The purification of oleuropein was achieved using a previously published protocol (Xynos et al. 2012).

All commercially available chemicals and solvents were purchased from Alfa Aesar and used as received without any further purification. Melting points were determined on a Büchi apparatus and were uncorrected. All NMR spectra (1D and 2D) were recorded on 400 or 600 MHz Bruker spectrometers respectively Avance™ DRX and III instruments (Bruker BioSpin GmbH — Rheinstetten, Germany). ^1^H-NMR (400 and 600 MHz) and ^13^C-NMR (101 and 151 MHz, recorded with complete proton decoupling) spectra were obtained with samples dissolved in CDCl_3_ or DMSO-*d*_6_ with the residual solvent signals used as internal references. Assignments of ^1^H and ^13^C-NMR signals were unambiguously achieved with the help of D/H exchange and 2D techniques: COSY, NOESY, HMQC, and HMBC experiments. Flash chromatography was performed on Merck silica gel (40–63 μm) with the indicated solvent system using gradients of increasing polarity in most cases (Merck KGaA — Darmstadt, Germany). The reactions were monitored by analytical thin-layer chromatography (Merck pre-coated silica gel 60 F254 TLC plates, 0.25-mm layer thickness). Compounds were visualized on TLC plates by both UV radiation (254 and 365 nm) and spraying with a staining agent (vanillin, PMA, KMnO_4_, or ninhydrin) followed by subsequent warming with a heat gun. All solvents for absorption and fluorescence experiments were of spectroscopic grade. Mass spectra were recorded on a hybrid LTQ™ Orbitrap Discovery XL instrument (Thermo Fisher Scientific — Bremen, Germany), coupled to an Accela HPLC system (Thermo Fisher Scientific) equipped with a binary pump, an autosampler, and Xcalibur 2.1 as a software.

### Cell culture

The human TNBC cell lines MDA-MB-231 and MDA-MB-468 were propagated, in Dulbecco’s modified Eagle medium (DMEM) with high glucose, supplied with 10% fetal bovine serum (FBS), 1% *L*-glutamine, 1% penicillin-streptomycin, and 1% non-essential amino acids, as proposed by ATCC®.

### Cytotoxicity screening

Cells were seeded at a concentration of 1 × 10^5^ cells/mL in a 96-well plate in triplicates of 100 μL. Oleuropein and oleocanthal in an initial concentration of 0.05 M in DMSO were serially diluted in complete cell culture medium, to create a concentration range of 50 μM–1 mM. For each different concentration, a volume of 350 μL was produced, so as to be enough for triplicates in a 96-well plate. The sulforhodamine B (SRB) assay was used for cell density determination, in 4 time points: 12, 24, 36, and 48 h after treatment. The SRB method has been demonstrated to provide reliable results and is widely recognized as a suitable approach for cell viability assessment (Keepers et al. 1991; Vichai and Kirtikara 2006).

Briefly, following the incubation period, cell monolayers were treated with a solution containing 10% trichloroacetic acid and stained for a duration of 60 min. The excess dye was then removed by repeatedly washing the cell monolayers with a solution containing 1% acetic acid. The rest of the dye, which was bound to the proteins, was then dissolved in a solution of 10 mM Tris base for optical density measurement at a wavelength of 510 nm, using a microplate reader (Vichai and Kirtikara 2006). Next, cell viability after treatment with each drug concentration was calculated as follows:

$$Cell\ viability=100-\left[ OD(sample)/ OD(control)\right]\ast 100$$

where “sample” stands for the average of the treated cells and “control” for the average of untreated cells.

### Total RNA extraction and RNA quality control

MDA-MB-231 and MDA-MB-468 cells were seeded in a 6-well plate and treated with the optimum oleuropein and oleocanthal concentrations that were previously determined. Cell viability was assessed again at each of the 4 aforementioned time points using the trypan blue exclusion test. Total RNA extraction was conducted in two replicates 12, 24, and 48 h after treatment from the treated and respective control (untreated) cells at each time point, using the NucleoZOL reagent (MACHEREY-NAGEL GmbH & Co. KG, Düren, Germany). The replicates were then combined. The concentration and integrity of the 12 total RNA extracts were assessed via electrophoresis in Tapestation 4150 (Agilent Technologies, Winooski, VT, USA).

### poly(A)-RNA enrichment, library construction, and RNA-seq

The NEBNext® Poly(A) mRNA Magnetic Isolation Module (New England Biolabs Ltd., Hitchin, UK) was utilized to isolate poly(A)-RNA from 1 μg of each total RNA extract. The concentration and length distribution of each poly(A)-RNA extract was assessed in Tapestation 4150 (Agilent Technologies). Subsequently, each poly(A)-enriched extract was used to produce a barcoded sequencing library, following the manufacturer’s instructions, with the use of the MGIEasy RNA Directional Library Prep Set (MGI Tech Co. Ltd., Shenzhen, China). The sequencing was carried out utilizing paired-end 100 chemistry of a DNBSEQ-G50RS High-throughput Sequencing Set, on the DNBSEQ-G50 platform (MGI Tech Co. Ltd.).

### RNA-seq data analysis

The NGS data was analyzed using the Partek® Flow® software, by conducting post-processing and bioinformatics analysis. Initially, low-quality bases were trimmed and quality control was conducted. All reads were then aligned to the human genome 38 (GRCh38) using the RNA STAR aligner (Dobin et al. 2013) (Fig. S1A). To determine gene expression, the reads were quantified to the Refseq transcripts. Due to the different total read count of each sequenced library, the counts were normalized to the total number of reads prior to differential expression analysis (Fig. S1B), to ensure sample comparability. Gene-set analysis (GSA) was used to determine differentially expressed genes (DEGs), by applying a fold-change cut-off of ± 1.5 and a *P* value threshold of < 0.050. Pathway enrichment analysis was conducted using R programming language, along with the enrichplot and DOSE packages (Yu 2023; Yu et al. 2015).

## Results

### Inhibitory effect of oleuropein and oleocanthal on TNBC cell lines

**Fig. 2:**
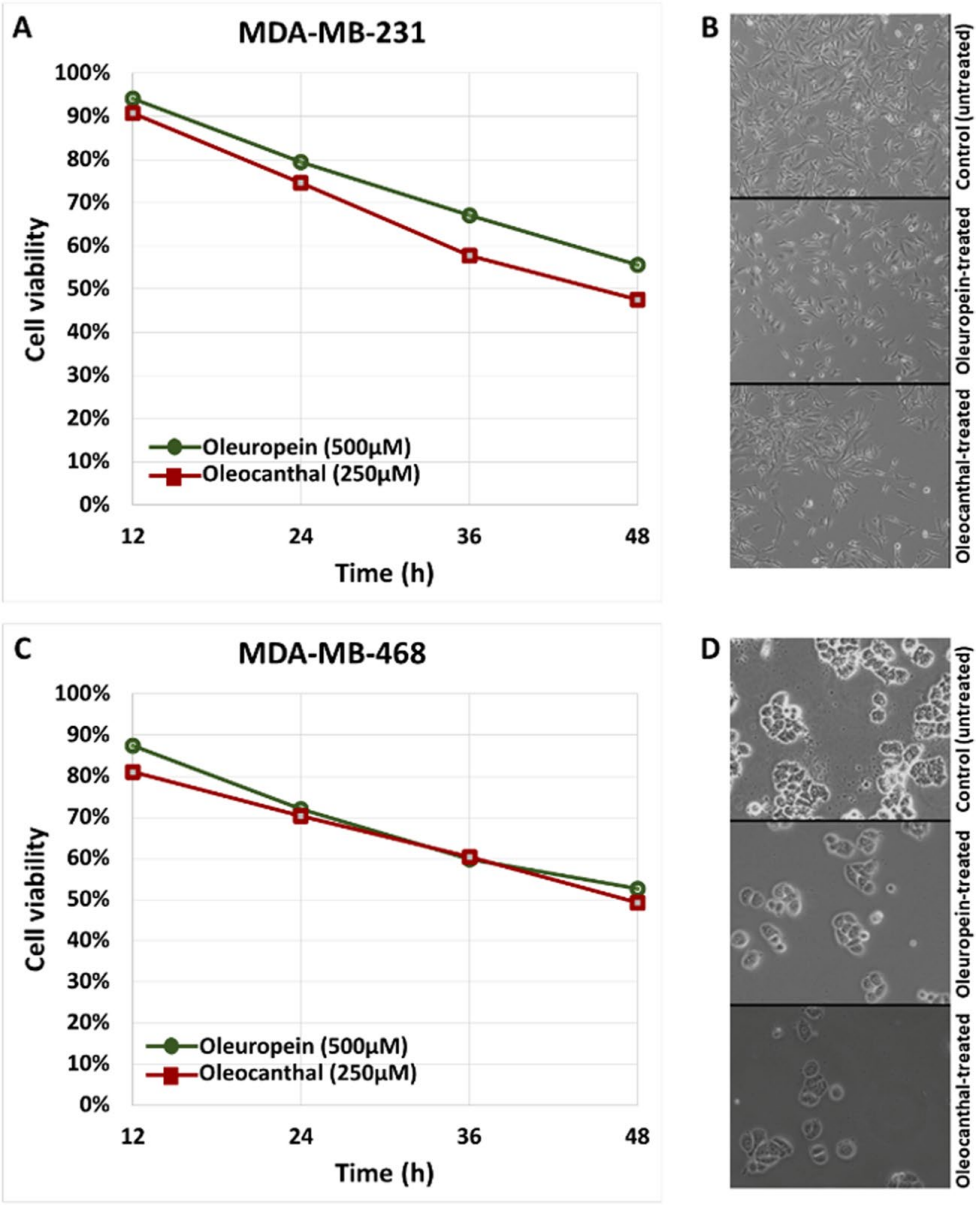
Illustration of % cell viability and imaging of triple-negative breast cancer cells 48 h after treatment with oleuropein or oleocanthal. In **A** and **B**, cell viability graph and imaging of MDA-MB-231 cells, respectively, are shown; **C** and **D** correspond to MDA-MB-468 cells. Cell viability was calculated using three biological replicates

The IC_50_ values 48 h after treatment with oleuropein or oleocanthal in MDA-MB-231 and MDA-MB-468 cell lines were determined by plotting % cell viability *vs.* time for each compound concentration that was tested, as revealed by the SRB assay. Oleuropein was found to have an IC_50_ of 500 μM in both MDA-MB-231 and MDA-MB-468 cells. Oleocanthal, on the other hand, exhibited an IC_50_ of 250 μM in both MDA-MB-231 and MDA-MB-468 cells (Fig. S2). Although the IC_50_ values are relatively high, other studies have also reported high IC_50_ values for these molecules, in both BrCa and other cancer cell lines (Asgharzade et al. 2020; Pastorio et al. 2022). These results suggest that both oleuropein and oleocanthal possess inhibitory effects on BrCa cell lines, with oleocanthal showing a slightly stronger effect than oleuropein. After determining the IC_50_ values, cells were treated with the selected concentration for each compound and visualized in an AxioVert.A1 vertical microscope (ZEISS, Oberkochen, Baden-Württemberg, Germany) Cell viability was again assessed after treatment with each optimum compound concentration and prior to RNA extraction (Fig. 2).

### Alteration of the expressional profile of TNBC cell lines after treatment with EVOO compounds

**Fig. 3:**
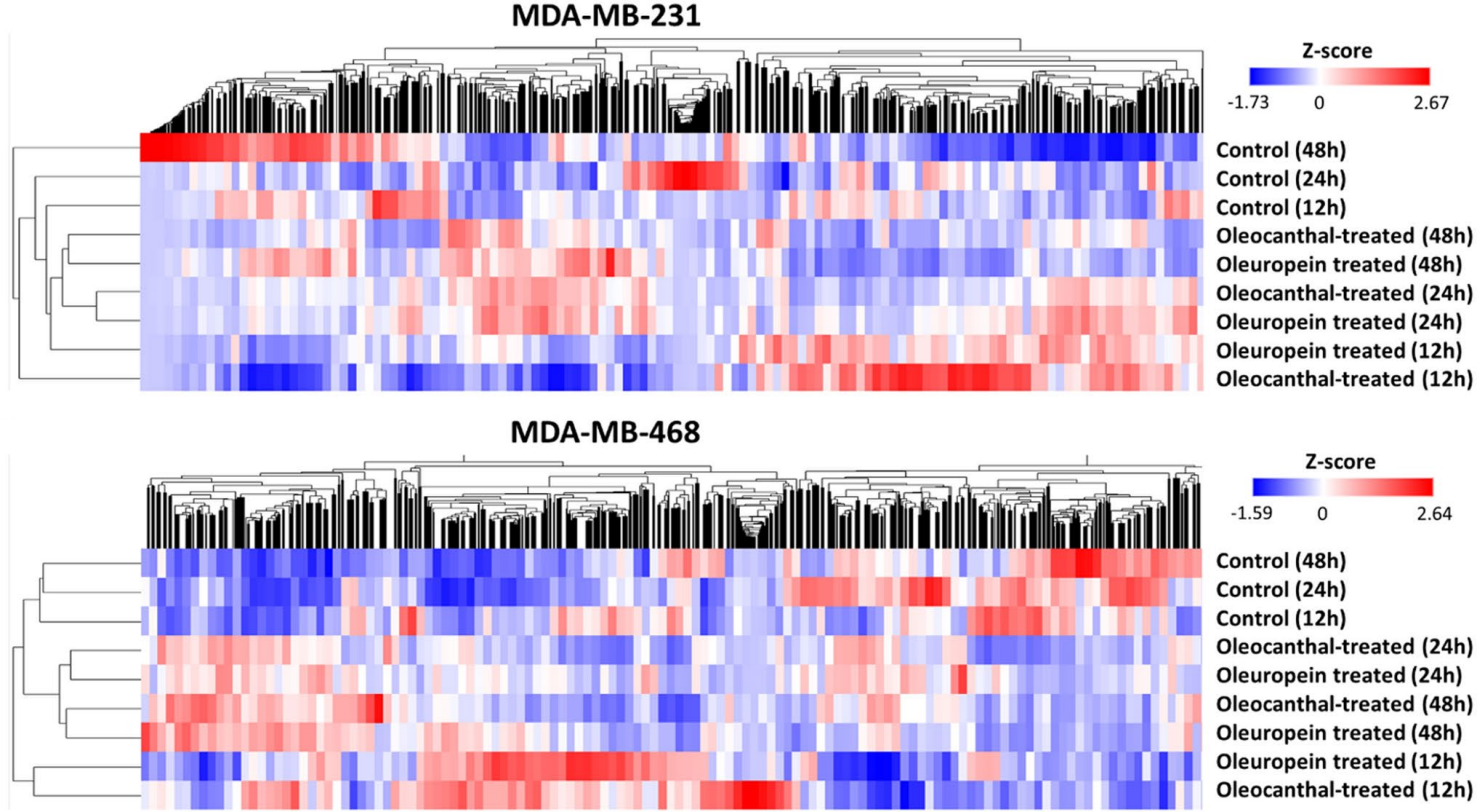
Hierarchical clustering heatmaps, showing the alterations in the gene expression profile of MDA-MB-231 and MDA-MB-468 cells at 3 time points after treatment with oleuropein or oleocanthal

As revealed by the NGS data analysis, the expression profile of both TNBC cell lines altered significantly as a result of treatment with either oleuropein or oleocanthal (Fig. 3). The maximum number of DEGs in MDA-MB-231 cells was found 48 h after oleuropein treatment (5898 genes), while the highest number was found 24 h after oleocanthal treatment (2900 genes). Oleuropein treatment had a common effect on 659 genes and oleocanthal treatment had a common effect on 565 genes in MDA-MB-231 cells across the 3 time points. Oleuropein treatment led to the greatest number of DEGs in MDA-MB-468 cells at 48 h (2887 genes), while oleocanthal treatment led to the greatest number at 24 h (2598 genes). Oleuropein therapy tended to consistently affect 768 genes, and oleocanthal treatment tended to consistently affect 822 genes among the time points assessed. These data demonstrate the important effects of oleuropein and oleocanthal on the gene expression profile of TNBC cell lines, with time-dependent gene expression changes and a group of genes that are consistently impacted at all time points (Table 1).

**Table 1:** The number of genes, the expression of which was affected by treatment of each breast cancer cell line with oleuropein or oleocanthal, at each time point

Number of genes affected after treatment						
Cell line	Compound	Treatment time interval			Common between the three time points	Common between the three time points and the two compounds
		12 h	24 h	48 h		
MDA-MB-231	Oleuropein	2995	3065	5898	659	148
	Oleocanthal	2891	2900	4750	565	
MDA-MB-468	Oleuropein	2402	1997	2887	768	496
	Oleocanthal	2514	2598	3021	822	

### Effect of oleuropein and oleocanthal treatment in pathways related to TNBC

**Fig. 4:**
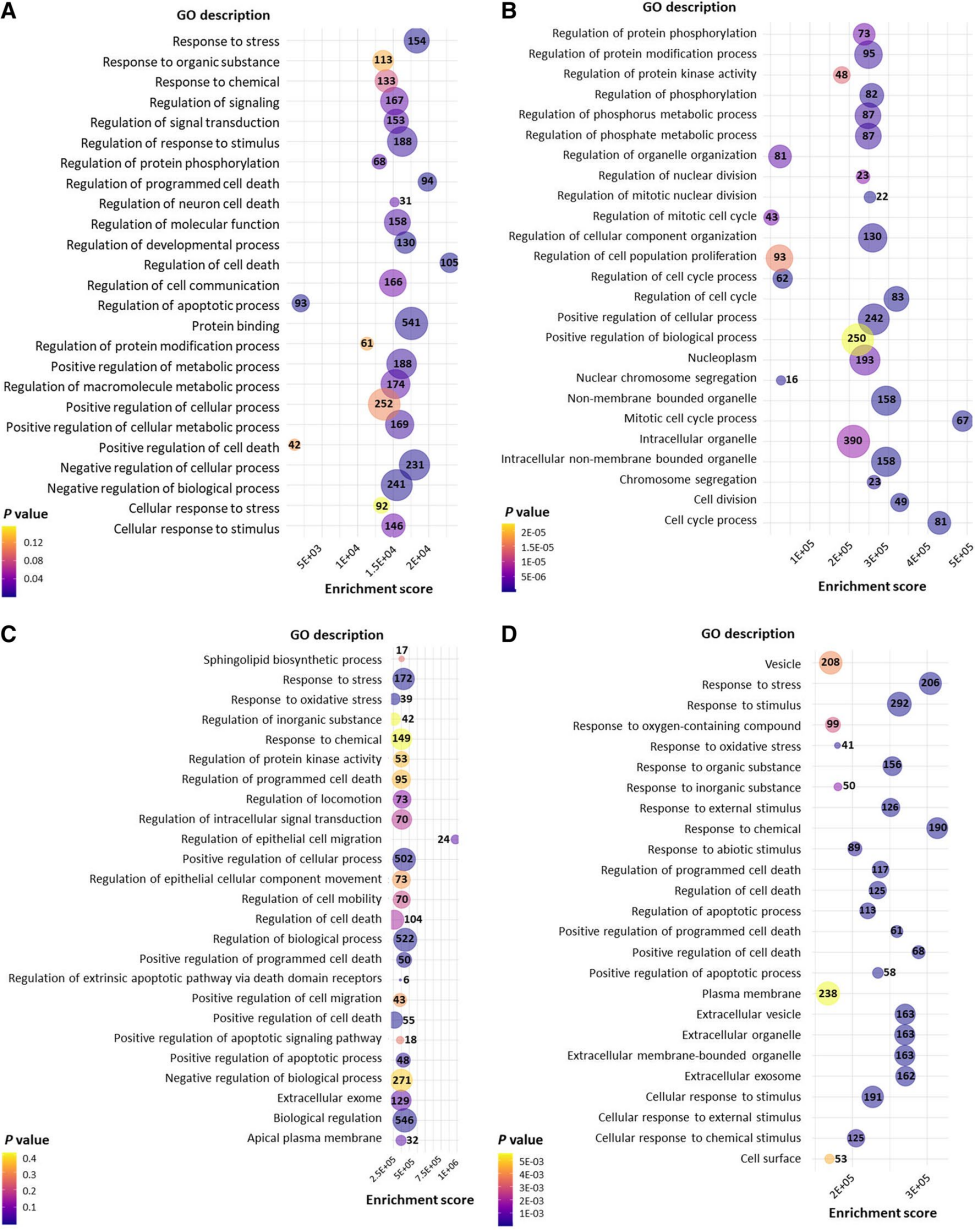

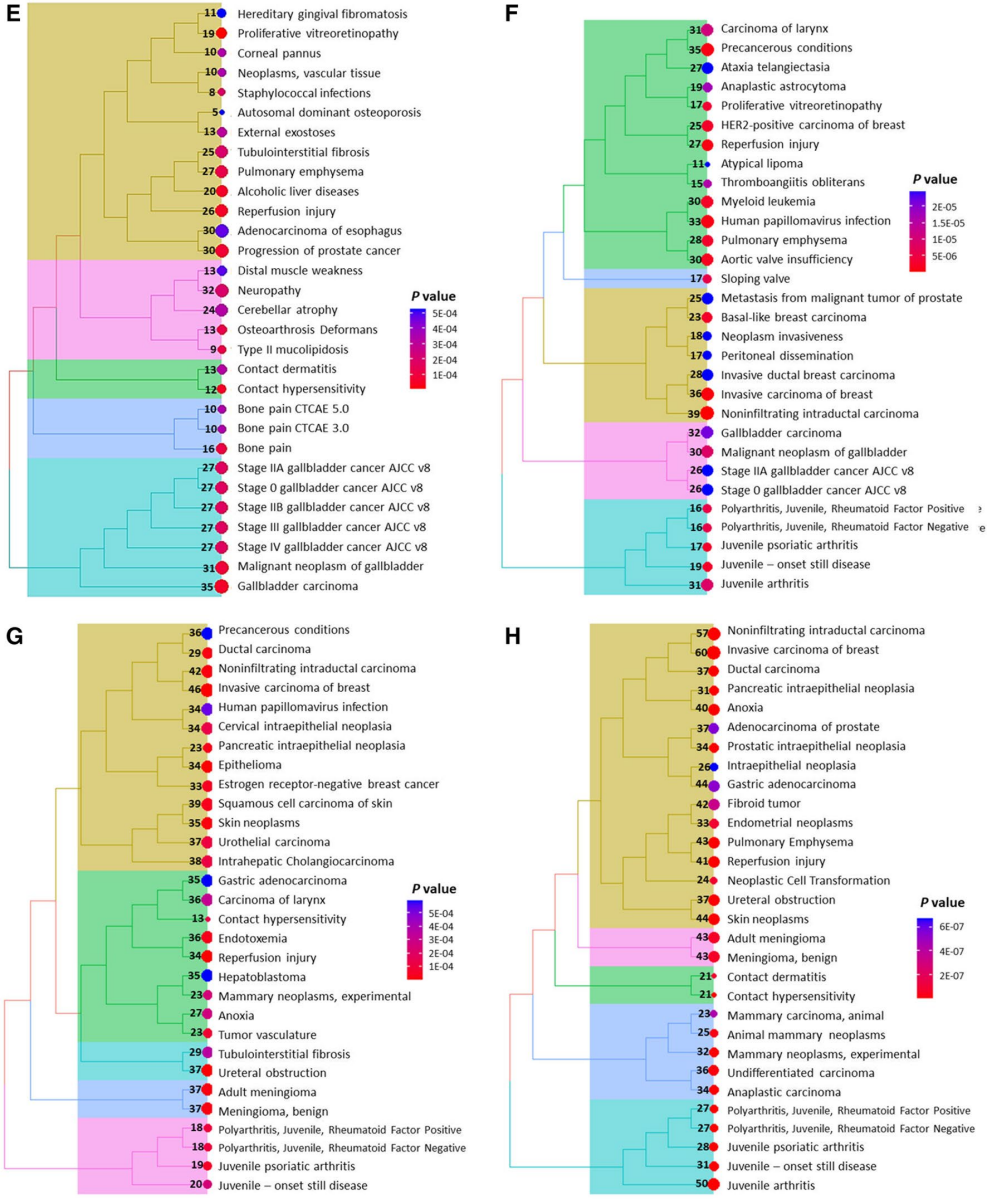
Illustration of the most significantly affected pathways upon treatment with natural products. The most significantly enriched GO processes after oleuropein and oleocanthal treatment in MDA-MB-231 cells are shown in **A** and **B** respectively, while in **C** and **D**, the respective ones for MDA-MB-468 cells are shown. Moreover, the most significantly enriched KEGG pathways after oleuropein and oleocanthal treatment in MDA-MB-231 cells are shown in **E** and **F** respectively, while in **G** and **H**, the respective ones for MDA-MB-468 cells are shown. The numbers in or next to the bubbles indicate the number of genes implicated in each pathway

The GO analysis of TNBC cells treated with oleuropein and oleocanthal revealed several significant results related to BrCa. The treatment with oleuropein in MDA-MB-231 cells showed enriched regulation of cell death, apoptotic process, programmed cell death, response to stress, protein binding, regulation of response to stimulus, and regulation of neuron death, among others (Harris et al. 2004). These findings suggest that oleuropein may potentially modulate cell death pathways in BrCa cells. Moreover, positive regulation of metabolic process and cellular metabolic process were observed, indicating potential effects on cellular metabolism in BrCa cells. In addition, treatment with oleocanthal in MDA-MB-231 cells showed regulation of the mitotic cell cycle process, cell division, chromosome segregation, regulation of protein phosphorylation, and regulation of cell population proliferation. These results suggest that oleocanthal may impact cell cycle regulation and cell proliferation in BrCa cells (Fig. 4A, B). In the MDA-MB-468 cells that were treated with both oleuropein and oleocanthal, regulation of biological processes, response to stress, and positive regulation of cell death were observed as significantly enriched, indicating potential effects of both compounds on BrCa cell viability and response to stress stimuli. Regulation of the apoptotic process, extracellular exosome, and response to chemical were also significantly enriched, suggesting a potential effect on apoptotic signaling and intercellular communication of these cells (Fig. 4C, D).

The KEGG pathway analysis identified several significant pathways associated with BrCa in both cell lines as well (Kanehisa et al. 2017). Pathways related to cancer progression, such as “Invasive carcinoma of breast” and “noninfiltrating intraductal carcinoma” were found, indicating the potential involvement of these pathways in the invasive and proliferative characteristics of BrCa cells. Furthermore, pathways related to cellular stress and damage, such as “reperfusion injury” and “anoxia” were also identified, indicating that BrCa cells may experience stress and damage in response to treatment with oleuropein and oleocanthal (Fig. 4E–H).

## Discussion

While TNBC is the most prevalent kind of BrCa in women globally, it has the fewest effective therapeutic options (Ferlay et al. 2021; Polyak 2011; Testa et al. 2020). Typically, TNBC does not respond to hormone treatments or targeted medications that target the three hormone receptors (estrogen, progesterone, and HER2 receptors). Chemotherapy is currently the standard of care for TNBC, but unfortunately, many patients eventually develop resistance to this type of treatment (Li et al. 2022). New and potent therapeutic approaches are thus urgently required for this BrCa subtype (Lehmann et al. 2011). Bioactive natural products have been used for medical purposes for a long time, and some of them have shown promise in treating a range of diseases, including cancer (Huang et al. 2021). TNBC natural product-based therapies have made tremendous strides in recent years (Kumar et al. 2023).

In this study, we examined how the EVOO compounds oleuropein and oleocanthal affected the viability of MDA-MB-231 and MDA-MB-468 TNBC cell lines. As shown by the IC_50_ values, both substances have inhibitory effects on these BrCa cell lines proliferative potential, with oleocanthal having a slightly stronger effect than oleuropein. The use of transcriptomics in this study allowed for a comprehensive analysis of the effects of oleuropein and oleocanthal on the expression profile of TNBC cell lines. Specifically, we discovered that treatment with either substance significantly alters the gene expression profile in TNBC cells, exhibiting distinct temporal gene expression dynamics and affecting a set of genes consistently across all time points.

The high concentration of bioactive chemicals in EVOO, such as oleocanthal, oleuropein, hydroxytyrosol, and tyrosol, is regarded to be the cause of the oil’s health advantages (Jimenez-Lopez et al. 2020). EVOO and its bioactive components have been the subject of numerous studies investigating possible anti-cancer properties in the treatment of BrCa. More specifically, the effects of oleuropein and oleocanthal treatment in cancer cell lines have been investigated before (Antoniou and Hull 2021). However, the majority of research has relied on protein-based assays to determine their effects, and only a small number have made use of transcriptomics, a potent tool that enables the investigation of a cell’s entire gene expression profile. Transcriptomics has the potential to shed light on the molecular processes that underlie the effects of oleuropein and oleocanthal on several cell types since its value in understanding the molecular mechanisms underlying novel treatment strategies has been variously highlighted before (Cui and Paules 2010; Yang et al. 2020). To completely comprehend the molecular pathways that these compounds influence and to find possible therapeutic targets for a variety of disorders, it is imperative that future studies use transcriptomics.

Another advantage of this work is the usage of a multi-time series time course experiment; in this kind of experiment, each time course dataset is directly compared to a control time course (Spies and Ciaudo 2015). Although this kind of study allows better control of the experiment, it is usually not preferred by researchers, due to the usage of multiple samples and consequently higher cost. Oleuropein treatment resulted in the highest number of DEGs in MDA-MB-231 cells at 48 h, whereas oleocanthal treatment resulted in the highest at 24 h. These results imply that depending on the particular cell line and experimental circumstances, the best time point to evaluate the gene expression changes brought on by oleuropein and oleocanthal treatment may vary. This emphasizes how crucial it is to pick the time points for transcriptome analysis properly (Kleyman et al. 2017; Spies and Ciaudo 2015), and how advantageous is the usage of a multi-time series time course experiment.

Interestingly, our findings on the inhibitory effects of oleuropein on TNBC cell lines echo results from prior studies. A comprehensive review by Ahmad Farooqi et al. outlined the multi-faceted role that oleuropein plays in modulating cellular pathways associated with cancer (Ahmad Farooqi et al. 2017). The authors highlighted oleuropein’s antioxidant, anti-inflammatory, and anti-proliferative properties, providing a broader biochemical context that complements our own observations. Specifically, several studies indicated that oleuropein not only inhibits the growth of cancer cells but also modulates key signaling pathways in several cancer types, including BrCa (Menendez et al. 2007; Menendez et al. 2008), which aligns with our findings of altered gene expression in TNBC cell lines treated with oleuropein.

Several relevant pathways associated with cancer progression, cellular stress and damage, and control of cell death were detected by pathway analysis. Our findings are in accordance with earlier research that demonstrated oleuropein’s anticancer activity in BrCa cell lines. For instance, oleuropein induces apoptosis via the intrinsic pathway in BrCa cells, as well as inhibits their proliferation by delaying the cell cycle at the S phase and upregulating p21 (Asgharzade et al. 2020; Elamin et al. 2013; Han et al. 2009). Oleuropein has also been demonstrated to prevent BrCa cells from migrating and invading by upregulating the expression of tissue inhibitors of matrix metalloproteinases (MMPs) and downregulating the expression of MMPs (Hassan et al. 2012). These findings are consistent with our GO analysis, which showed that oleuropein treatment of TNBC cells enriched pathways involved in apoptosis, cell death, and cell cycle. Moreover, oleuropein has been reported to alter the expression of apoptosis-related genes at mRNA level in MDA-MB-231 and MDA-MB-468 cells, as revealed by quantitative real-time PCR experiments (Messeha et al. 2020), and of histone deacetylases in MCF-7 cells (Mansouri et al. 2019).

Another interesting result observed upon oleuropein treatment was its effect on metabolic processes. TNBC is characterized by metabolic reprogramming, related to its molecular features (Sun et al. 2020), while oleuropein has been shown to affect some metabolic pathways (Polzonetti et al. 2004). Since this axis has been poorly investigated, it would be interesting to explore such an effect of *Olea europaea* natural products in future research.

According to our results, oleocanthal inhibits cancer cells’ viability more potently than oleuropein does, which is in line with earlier research (Papakonstantinou et al. 2023). In line with our observations on the effects of oleocanthal on TNBC cell lines, previous research has also emphasized the anti-cancer properties of this bioactive compound. A study by LeGendre et al. delved into the molecular mechanisms by which oleocanthal induces apoptosis in cancer cells (LeGendre et al. 2015). The study identified oleocanthal’s unique ability to disrupt cancer cell lysosomes, leading to cell death. These insights contribute to a broader understanding of how oleocanthal might affect TNBC cells, particularly given our findings that oleocanthal alters gene expression and enriches specific cellular pathways in these cells. Although this compound has not been thoroughly investigated in BrCa, it has been reported to generally exert anti-BrCa activity (Siddique et al. 2019); regarding TNBC, it has been shown to reduce MDA-MB-231 cell viability and hinder migration and proliferation (Diez-Bello et al. 2019; Qusa et al. 2021), confirming our results about the implication of oleocanthal in cell death processes. Treatment of MDA-MB-231 cells with oleocanthal has been shown to lead to reduced phosphorylated mTOR levels (Khanfar et al. 2015), a finding that comes in line with our GO analysis, showing that treatment with oleocanthal affected protein phosphorylation and regulation of protein kinase activity. Similarly, oleocanthal has also been shown to arrest the cell cycle at the G1/S phase in MDA-MB-231 cells (Akl et al. 2014), explaining the enriched processes related to the cell cycle.

It is important to note the limitations of the study we conducted. The potential anti-cancer effects of oleuropein and oleocanthal in TNBC patients need to be confirmed in additional research utilizing in vivo models and/or clinical trials, since our findings are based on in vitro cell line experiments. Moreover, our KEGG pathway analysis provided evidence that TNBC cells experienced stress and damage upon treatment with the bioactive compounds; this fact could imply undesired side effects that could only be investigated in vivo. Second, further investigation is required to clarify the processes underlying the effects of oleuropein and oleocanthal on cellular pathways and gene expression. Future research could focus on determining the molecular processes and signaling pathways through which oleuropein and oleocanthal inhibit TNBC cell growth. Moreover, since a large list of genes affected by the treatment with these natural products was generated, future research could focus on specific genes to examine as direct therapeutic targets of these compounds.

In conclusion, our study demonstrates that oleuropein and oleocanthal have anti-cancer effects in TNBC cell lines by inhibiting cell survival and changing the gene expression profile of these cells. Additionally, according to our findings, these substances may affect several pathways involved in the development of cancer, cellular metabolism, and stress response. In further studies, researchers should carefully choose the time points for transcriptome analysis because of the distinctive temporal dynamics of the gene expression alterations brought on by oleuropein and oleocanthal therapy. Overall, our findings add to the growing body of research that suggests EVOO compounds may be therapeutically useful in treating BrCa, particularly TNBC.

### Supplementary information

ESM 1 (DOCX 1276 kb)

## Data Availability

Our data are not deposited in publicly available repositories. However, the datasets used and/or analyzed during the current study are available from the corresponding author upon reasonable request.
