# Supplementary material for: Next-generation sequencing reveals altered gene expression and enriched pathways in triple-negative breast cancer cells treated with oleuropein and oleocanthal
Source: Funct Integr Genomics. 2023 Sep 14;23(4):299. doi: 10.1007/s10142-023-01230-w (PMC10501944; doi:10.1007/s10142-023-01230-w)
Supplement: Supplementary file 1 — (DOCX 1276 kb) [file 10142_2023_1230_MOESM1_ESM.docx]

**Supplementary material**

**Experimental**

All commercially available chemicals and solvents were purchased from Alfa Aesar and used as received without any further purification. Melting points were determined on a Büchi apparatus and were uncorrected. All NMR spectra (1D and 2D) were recorded on 400 or 600 MHz Bruker spectrometers respectively Avance™ DRX and III instruments (Bruker BioSpin GmbH – Rheinstetten, Germany). ^1^H-NMR (400 and 600 MHz) and ^13^C-NMR (101 and 151 MHz, recorded with complete proton decoupling) spectra were obtained with samples dissolved in CDCl_3_ or DMSO‐*d*_6_ with the residual solvent signals used as internal references. Assignments of ^1^H and ^13^C-NMR signals were unambiguously achieved with the help of D/H exchange and 2D techniques: COSY, NOESY, HMQC, and HMBC experiments. Flash chromatography was performed on Merck silica gel (40–63 *μ*m) with the indicated solvent system using gradients of increasing polarity in most cases (Merck KGaA – Darmstadt, Germany). The reactions were monitored by analytical thin-layer chromatography (Merck pre-coated silica gel 60 F254 TLC plates, 0.25-mm layer thickness). Compounds were visualized on TLC plates by both UV radiation (254 and 365 nm) and spraying with a staining agent (vanillin, PMA, KMnO_4_ or ninhydrin) followed by subsequent warming with a heat gun. All solvents for absorption and fluorescence experiments were of spectroscopic grade. Mass spectra were recorded on a hybrid LTQ™ Orbitrap Discovery XL instrument (Thermo Fisher Scientific – Bremen, Germany), coupled to an Accela HPLC system (Thermo Fisher Scientific) equipped with a binary pump, an autosampler, and Xcalibur 2.1 as a software.

*Synthesis of 4-(2-hydroxyethyl)phenyl acetate*

Tyrosol (500 mg, 0.03 mol, 1 equiv) was dissolved in an aqueous medium (4 mL, 70 equiv) and a aqueous NaOH (0.11 mol, 3 equiv, 2 mL, pH 7-8) solution was gradually added at 0°C. The reaction’s temperature was controlled at 0°C using an ice bath. After completion of the reaction (20 min), as indicated by TLC monitoring (cyclohexane− EtOAc 1:1), the product was extracted with CH_2_Cl_2,_ dried (anhydrous Na_2_SO_4_), and concentrated to dryness to afford crude 2-(4-acetoxyphenyl)ethanol. The crude product was purified by flash chromatography (silica gel), using CH_2_Cl_2_/MeOH, 100 → 99:1 v/v as the eluent and 2-(4-acetoxyphenyl)ethanol was obtained in 82% yield. ^1^H NMR (600 MHz, CDCl_3_) δ: 7.19 (d, 2H), 6.99 (d, 2H), 3.75 (t, 2H), 2.80 (t, 2H), 2.25 (s, 3H).

*Synthesis of Oleoside or (4S,5E,6S)-4-(carboxymethyl)-5-ethylidene-6-[(2S,3R,4S,5S,6R)-3,4,5-trihydroxy-6-(hydroxymethyl)oxan-2-yl]oxy-4H-pyran-3-carboxylic acid (****1****)*

To a solution of oleuropein (11 g, 20.4 mmol) in H_2_O (150 mL), at room temperature, a cold NaOH solution (3.25 g. 81.4 mmol in 50 mL of H_2_O) was added dropwise. The resulting mixture was stirred for 20 hrs at room temperature, acidified with 2N HCl (pH ~ 4.5), concentrated under reduced pressure and purified by Centrifugal Partition Chromatography (CPC) to afford 7.42 g (90%) of the title compound. The centrifugal system of solvents was EtOAc / 2-Propanol / EtOH / H_2_O / Acetic acid 8 / 2/ 1 / 10 / 0.5 (8.6 L), with a column capacity of 1L, rapid rotation at 800 rpm, and a flow rate 15 mL / min. Rf 0.20 (CH_2_Cl_2_−MeOH 6:4). ^1^H NMR (600 MHz, D_2_O) *δ* 7.68 (s, 1H, H-3), 6.25 (q, *J*_8,10_ = 7.09 Hz, 1H, H-8), 6.04 (s, 1H, H-1), 5.04 (d, *J*_1′,2′_ = 8.03 Hz, 1H, H-1′), 4.11 (dd, *J*_5,6b_ = 9.63, *J*_5,6a_ = 4.57 Hz, 1H, H-5), 4.01 (dd, *J*_6a′,6b′_ = 12.49, *J*_6a′,5′_ = 2.20 Hz, 1H, H-6a′), 3.83 (dd, *J*_6b′,6a′_ = 12.49, *J*_6b′,5′_ = 5.92 Hz, 1H, H-6b′), 3.65-3.62 (m, 1H, H-3′), 3.61 (ddd, *J*_5′,4′_ = 8.20, *J*_5′,6b′_ = 5.92, *J*_5′,6a′_ = 2.20 Hz 1H, H-5′), 3.54-3.52 (m, 1H, H-4′), 3.52-3.50 (m, 1H, H-2′), 2.91 (dd, *J*_6a,6b_ = 13.67, *J*_6a,5_ = 4.57 Hz, 1H, H-6a), 2.54 (dd, *J*_6b,6a_ = 13.67, *J*_6b,5_ = 9.63 Hz, 1H, H-6b), 1.84 (dd, *J*_10,8_ = 7.12, *J*_10,1_ = 1.30 Hz, 3H, H-10); ^13^C NMR (151 MHz, D_2_O) δ 176.47 (C-7), 170.50 (C11), 154.62 (C-3), 128.46 (C-9), 125.06 (C-8), 108.68 (C-4), 99.73 (C-1′), 94.96 (C-1), 76.42(C5′), 75.74 (C-3′), 72.73(C-2′), 69.55 (C-4′), 60.71 (C-6′), 40.31 (C-6), 30.70(C-5), 12.88 (C-10); HRMS (ESI-) m/z 389.1091 (calcd for C_16_H_21_O_11_ 389.1089).

*Synthesis of* *Demethyl ligstroside or (2S,4S,E)-3-ethylidene-4-(2-(4-hydroxyphenethoxy)-2-oxoethyl)-2{[(2R,3S,4R,5R,6S)-3,4,5-trihydroxy-6-(hydroxymethyl)tetrahydro-2H-pyran-2-yl]oxy}-3,4dihydro-2H-pyran-5-carboxylic acid (****4****)*

Acetic anhydride (3.3 mL, 34.99 mmol, 17.5 equiv.) was added to a solution of **1** (808 mg, 2 mmol, 1 equiv.) in pyridine (1.56 mL, 19.99 mmol, 10 equiv.) at 0°C, and the resulting mixture was stirred at room temperature (rt) for 2 h, under argon. After completion of the reaction, as indicated upon TLC monitoring, the mixture was concentrated under reduced pressure to afford anhydride **2**. Crude **2**, without any further purification, dissolved in dry acetonitrile (5 mL) and 4-(2-hydroxyethyl)phenyl acetate (152 mg, 0.845 mmol, 1.1 equiv.), triethylamine (285.73 µL, 2.05 mmol, 2 equiv.), and DMAP (125 mg, 1.02 mmol, 1 equiv.) were added. The resulting solution was stirred at r.t. for 3 h, acidified with 2 N HCl (pH ~ 4–5), and vacuum-evaporated. The residue was dissolved in CH_2_Cl_2_, washed with water, dried (Na_2_SO_4_), and concentrated to dryness to afford crude **3**. Without further purification, the residue was dissolved in ethanol (85% aqueous) (5 mL) and diethylamine (364 *μ*L, 7.5 mmol, 6 equiv.) was added to the solution. The resulting solution was stirred for 6 h at 44°C, acidified with HCl 9% (pH ~ 4–5), and evaporated to dryness. The residue was dissolved in EtOAc, washed with water, dried (Na_2_SO_4_), and concentrated to dryness. The crude product was purified by flash chromatography (silica gel), using CH_2_Cl_2_/MeOH, 100 → 85:15 v/v as the eluent, to afford the title compound **4** (530 mg, 52%). Rf 0.35 (CH_2_Cl_2_−MeOH 8:2). ^1^H NMR (600 MHz, D_2_O): *δ* 7.58 (s, 1H, H-3), 7.25 (d, *J*_4′,5′/8′,7′_ = 7.74 Hz, 2H, H-4′/H-8′), 6.94 (d, *J*_5′,4′/7′,8′_ = 7.85 Hz, 2H, H-5′/H-7′), 6.12 (q, *J*_8,10_ = 6.85 Hz, 1H, H-8), 5.86 (s, 1H, H-1), 4.96 (d, *J*_1′′,2′′_ = 8.04 Hz, 1H, H-1′′), 4.39 (dt, *J*_1a′,1b′_ = 10.52 Hz, *J*_1a′,2′_ = 6.73 Hz, 1H, H-1a′), 4.27 (dt, *J*_1b′,1a′_ = 10.56 Hz , *J*_1b′,2′_ = 6.14 Hz, 1H, H-1b′), 3.98 (dd, *J*_6a′′,6b′′_ = 12.38, *J*_6a′′,5′′_ = 1.68 Hz, 1H, H-6a′′), 3.97-3.96 (m, 1H, H-5), 3.82 (dd, *J*_6b′′,6a′′_ = 12.33, *J*_6b′′,5′′_ = 5.51 Hz, 1H, H-6b′′), 3.65-3.62 (m, 1H, H-3′′), 3.57 (ddd, *J*_5′′,4′′_ = 10.02, *J*_5′′,6b′′_ = 5.41, *J*_5′′,6a′′_ = 1.89 Hz 1H, H-5′′), 3.55-3.52 (m, 1H, H-4′′), 3.52-3.50 (m, 1H, H-2′′), 2.95 (t, *J*_2′,1a′,1b′_ = 5.98 Hz, 2H, H-2′), 2.78 (dd, *J*_6a,6b_ = 13.81, *J*_6a,5_ = 4.75 Hz, 1H, H-6a), 2.56 (dd, *J*_6b,6a_ = 13.57, *J*_6b,5_ = 8.95 Hz, 1H, H-6b), 1.66 (d, *J*_10,8_ = 7.10 Hz, 3H, H-10); ^13^C NMR (151 MHz, D_2_O): *δ* 174.19 (C-7), 170.78 (C-11), 154.08 (C-3), 130.37 (C-3′/C-4′/C-8′), 128.51 (C-9), 124.98 (C-8), 115.51 (C-5′/C-7′), 109.07 (C-4), 99.57 (C-1′′) , 94.78 (C-1), 76.38 (C-5′′), 75.76 (C-3′′), 72.77 (C-2′′), 69.44 (C-4′′), 66.36 (C-1′), 60.77 (C-6′′), 39.87 (C-6), 33.29 (C-2′), 30.53 (C-5), 12.68 (C-10); HRMS (ESI-) m/z 509.1670 (calcd for C_24_H_29_O_12_ 509.1664).

*Synthesis of* *Oleocanthal or (3S,4E)-4-formyl-3-(2-oxoethyl)-4-hexenoic acid 2-(4-hydroxyphenyl)ethyl ester*

A mixture of compound **4** (510 mg, 1 mmol, 1 equiv) in HCl 1N (25 ml) and cyclohexane (25 ml) was stirred vigorously for 7 days at 28°C. After completion of the reaction, the water solution was washed with cyclohexane, dried (anhydrous Na_2_SO_4_), and concentrated to dryness. The residue was purified by flash chromatography (silica gel) using a mixture of CH_2_Cl_2_/MeOH, 100 → 95:5 v/v, to obtain oleocanthal (198 mg, 65%). Rf 0.52 (c-hex−EtOAc 2:3). ^1^H NMR (600 MHz, CDCl_3_) *δ* 9.62 (brs, 1H, H-3), 9.23 (d, *J*_1,5_ = 1.97 Hz, 1H, H-1), 7.04 (d, *J*_4′,5′/8′,7′_ = 8.42 Hz, 2H, H-4′/H-8′), 6.76 (d, *J*_5′,4′/7′,8′_ = 8.45 Hz, 2H, H-5′/H-7′), 6.65 (q, *J*_8,10_ = 7.08 Hz, 1H, H-8), 4.24 (dt, *J*_1a′,1b′_ = 10.86, *J*_1a′,2′_= 6.99 Hz, 1H, H-1a′), 4.20 (dt, *J*_1b′,1a′_= 10.77, *J*_1b′,2′_= 6.97 Hz, 1H, H-1b′), 3.63–3.58 (m, 1H, H-5), 2.99 (ddd, *J*_4a,4b_ = 18.26, *J*_4a,5_ = 8.61, *J*_4a,3_ = 1.16 Hz, 1H, H-4a), 2.83 (t, *J*_2′,1′_ = 6.92 Hz, 2H, H-2′), 2.75 (dd, *J*_4b,4a_ = 18.33, *J*_4b,5_ = 5.64 Hz, 1H, H-4b), 2.70 (dd, *J*_6a,6b_ = 15.87, *J*_6a,5_ = 8.26 Hz, 1H, H-6a), 2.63 (dd, *J*_6b,6a_ = 15.91, *J*_6b,5_ = 6.66 Hz, 1H, H-6b), 2.07 (d, *J*_10,8_ = 7.06 Hz, 3H, H-10); ^13^C NMR (151 MHz, CDCl_3_) *δ* 200.62 (C-3), 195.32 (C-1), 172.13 (C-7), 154.64 (C-6′), 154.45 (C-8), 143.49 (C-9), 130.15 (C-3′), 129.82 (C-4′/C-8′), 115.55 (C-5′/C-7′), 65.34(C-1′), 46.36 (C-4), 37.07 (C-6), 34.32 (C-2′), 27.45 (C-5), 15.35 (C-10); HRMS (ESI+) m/z 327.1200 (calcd for C_17_H_20_O_5_Na 327.1203).

**Supplementary Figures**


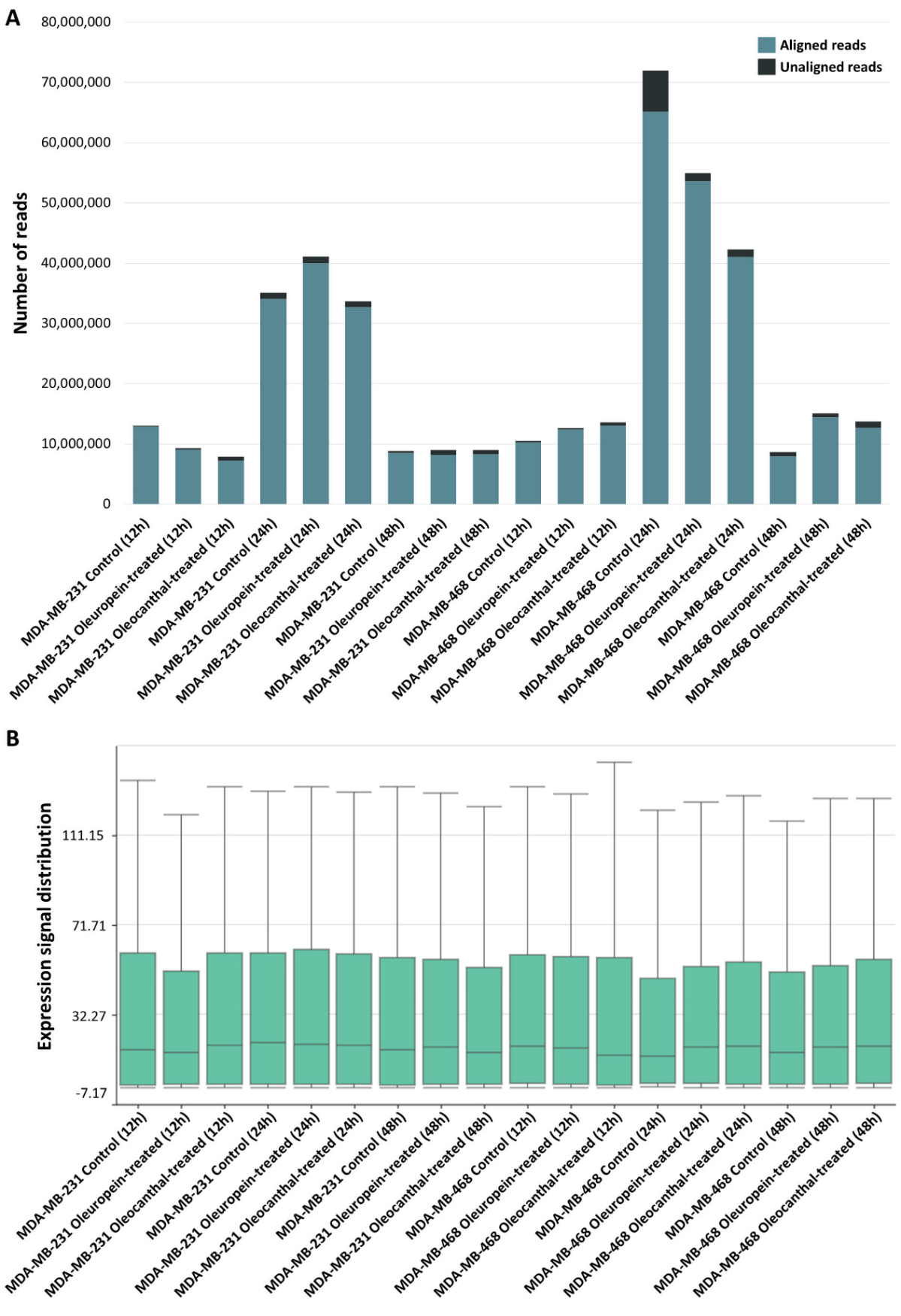


**Fig. S1** Graphic representation of the number of reads generated during the sequencing run **(A)** and of the gene expression counts after normalization **(B)**.


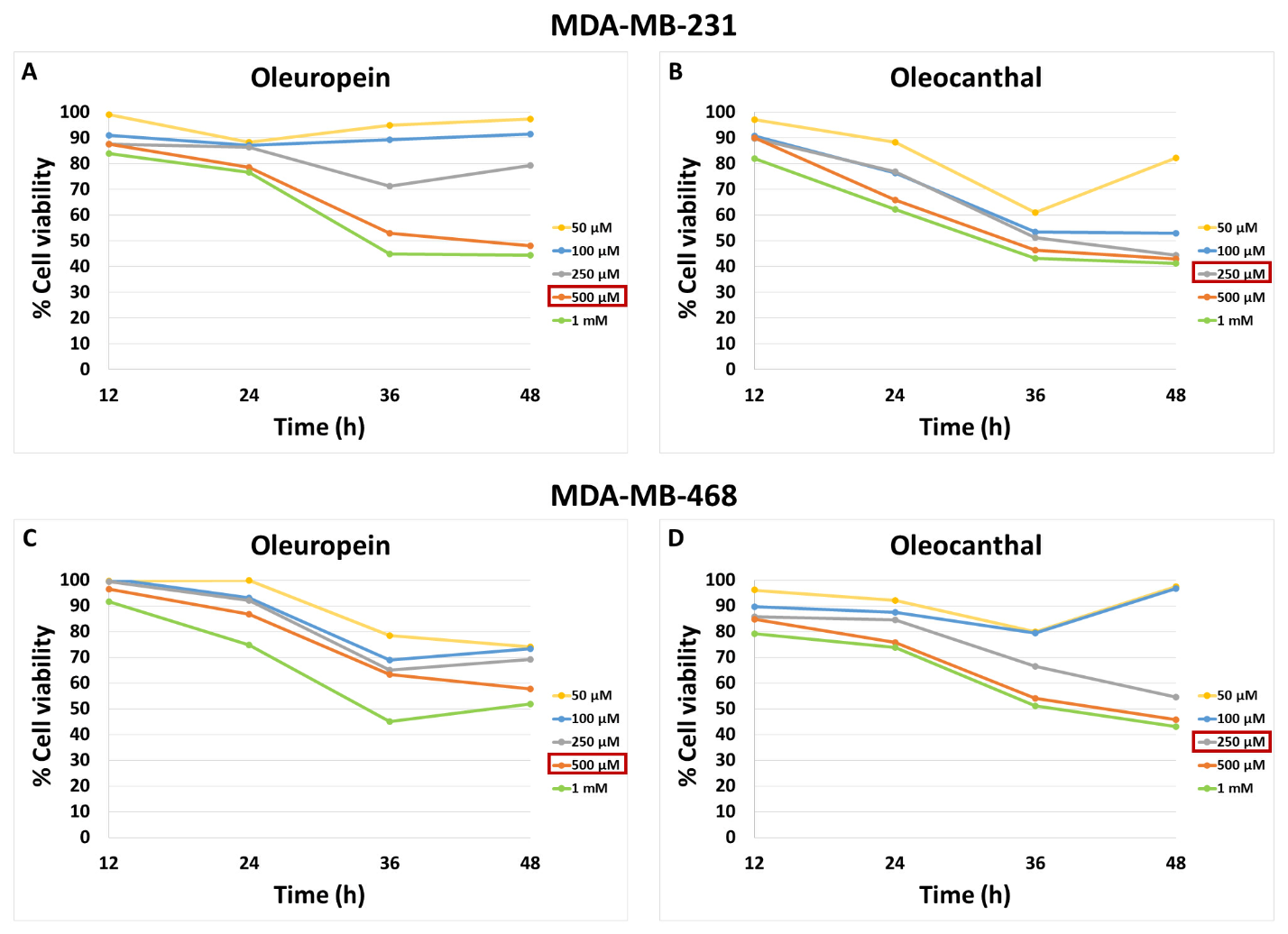


**Fig. S2** Illustration of % cell viability of MDA-MB-231 cells after treatment with a concentration range of oleuropein **(A)** and oleocanthal **(B)**, and of MDA-MB-468 cells after treatment with a concentration range of oleuropein **(C)** and oleocanthal **(D)**. Cell viability was calculated using three biological replicates.
